# Can the Physical Development Trajectories of Rugby League Players at Different Age Groups Inform the Talent Pathway? A Multi‐Club Study of 261 Players

**DOI:** 10.1002/ejsc.70100

**Published:** 2025-12-08

**Authors:** Sam Wild, Cameron Owen, Ben Jones, Sean Scantlebury, Paul Anderson, John Alder, Kevin Till

**Affiliations:** ^1^ Carnegie Applied Rugby Research (CARR) Centre Carnegie School of Sport Leeds Beckett University Leeds UK; ^2^ England Performance Unit Rugby Football League Manchester UK; ^3^ Premiership Rugby London UK; ^4^ Division of Physiological Sciences, Department of Human Biology, Faculty of Health Sciences University of Cape Town Cape Town South Africa; ^5^ Faculty of Health Sciences School of Behavioural and Health Sciences Australian Catholic University Brisbane Australia; ^6^ Welsh Rugby Union Cardiff UK; ^7^ Leeds Rhinos Rugby League Club Leeds UK

**Keywords:** analysis, performance, talent, team sport, youth

## Abstract

The structure of a talent identification and development system (TIDS), in terms of its starting, entry, and exit points is an important consideration for sporting organisations. Early talent identification decisions can be ineffective due to unpredictable and individually variable talent development. Physical qualities are a key contributor to performance in rugby league. Therefore, understanding physical development differences between age groups can inform the structure of the rugby league TIDS by highlighting key phases of development. Between‐player variability in physical development must also be considered to understand the generalisability of age‐group trends. Consequently, this study aimed to compare rates of physical development between annual age groups (i.e., U15, 16, 17, 18) in 261 youth rugby league players from multiple clubs, considering individual differences in development rates. Latent growth curve analysis was used to model rates of physical development for size (i.e., height, mass), strength, power, speed, and cardiovascular fitness in each age group. Results showed that U15s had significantly faster rates of development for body size and strength qualities compared with all older age groups, with large between‐player variability. No differences were apparent between age groups for power, speed, or cardiovascular fitness. These findings suggest that early talent identification and (de)selection decisions may ignore the potential development of body size and strength qualities, which occurs at individually variable rates. Such findings can inform the structure and design of the rugby league TIDS by highlighting expected rates of physical development based on players' age groups.

## Highlights

- Under‐15s developed body size and strength qualities at a faster rate than players in older age groups.
- No age‐group differences seen in rates of development for CMJ height, sprint speed, or cardiovascular fitness.
- Large between‐player variability evident in the development of body size.
- Early talent identification decisions in rugby league may not account the physical development which takes place in younger age groups.

## 1 Introduction

Talent identification and development systems (TIDS) are designed to facilitate the transition of talented youth athletes to the elite level of their sport. Successful transitions can be challenging given that talent is considered individually variable and emergent (Baker et al. 2019; Till and Baker 2020). This means talent identification is typically more effective in older youth athletes, questioning early talent identification practices (Abbott et al. 2005; Bailey and Collins 2013). Consequently, the point at which talent identification processes begin within a TIDS should be carefully considered. Following initial talent identification, ongoing talent (de)selection decisions are often required within TIDS (Baker et al. 2018). This is due to the need to concentrate limited resources in supporting the development of smaller numbers of athletes as they progress closer to the elite level (Bailey and Collins 2013). Making talent selection decisions early can also limit their effectiveness (Baker et al. 2018), meaning the structure of a TIDS, in relation to its starting, entry, and exit points, is an important consideration for effective talent identification and selection.

The professional rugby league TIDS in England begins at the Under‐15 (U15) age group and continues to U18s. Following this, players can continue towards either senior professional, senior semi‐professional, or reserve‐grade (semi‐professional within a professional club) rugby league. Players are organised into bi‐annual age groups who train and play together, with entry and exit points available within each annual age group. As with other sports, the commencement of rugby league's TIDS has previously been delayed (i.e., from U13s to U15s) but debate about the ideal start point still exists (Till and Bell 2019). Currently within the rugby league TIDS, initial talent identification occurs at the U14 age group, with ongoing talent (de)selection decisions at each annual age group up to U18s. Making these decisions in earlier age groups increases the risk of deselecting or not identifying potentially talented players based on their current performance without considering their potential future development (Bailey and Collins 2013). As such, understanding age group related differences in player development can inform the timing of these decisions, by highlighting the degree of potential physical development which may take place within each age group.

Rugby league match‐play is characterised by intermittent periods of repeated high‐intensity physical activity involving actions such as sprinting and tackling (Gabbett et al. 2011b; Johnston et al. 2014). The demands of the sport dictate that physical qualities increase with age (Gabbett 2002; Till et al. 2012, 2014), discriminate between playing levels (Gabbett 2009; Gabbett et al. 2010; Ireton et al. 2019; Pearce et al. 2018), and relate to match performance (Gabbett et al. 2007; Johnston et al. 2015; Speranza et al. 2015, 2016). Furthermore, evidence shows that physical qualities are a significant predictor of career attainment in youth players, suggesting that these qualities influence talent identification and selection decisions within TIDS (Till, Jones, et al. 2016). Age‐group based differences in rates of development for a range of physical qualities including strength, body size, lower body power, and cardiovascular fitness have been evidenced previously in U15‐U20 players (Till et al. 2015, 2014). This emphasises the importance of the timing of talent identification and selection decisions to account for players' potential development in rugby league. However, this work assessed players at one time point per season, featuring players from a single club; therefore, the findings do not account for varying rates of change within a season, or provide results that are generalisable to players at other clubs within the TIDS (Till et al. 2015, 2014). Furthermore, it should be considered that physical development appears to occur at individually variable rates (Till et al. 2013), making it difficult to predict. This suggests that further work is needed to build on existing research and establish whether age‐group based differences in rates of physical development are present in a wider sample of players within the rugby league TIDS, whilst considering between‐player variability in rates of development. These findings can better inform the timing of talent identification and selection decisions to ensure more effective decision making.

Overall, understanding differences in physical development between age groups, across multiple clubs, can inform the timing of talent identification and selection decisions in the rugby league TIDS. Early (de)selection and talent identification create a risk of losing talented players through ineffective decision making, particularly as rates of development can be individually variable (Till et al. 2013, 2022). As such, the aim of this study was to compare the rates of physical development between annual age groups (i.e., U15, 16, 17, 18) in youth rugby league players, considering individual differences in development rates. This can help inform the structure of the TIDS in relation to the timing of selection decisions and therefore how effectively the TIDS can transition players to the senior professional level.

## 2 Materials & Methods

### 2.1 Design

Physical fitness testing was conducted by the lead researcher across the professional rugby league TIDS in England throughout the 2023 and 2024 seasons. Testing occurred at four time‐points each season; start of pre‐season (October‐November), end of pre‐season (January–February), mid‐season (May–July), and end of season (August–September). This study included data from 261 youth male rugby league players, across six clubs and four annual age groups; U15 (*n* = 52), U16 (*n* = 46), U17 (*n* = 68), and U18 (*n* = 44). Participants were only included in the analysis if they were tested three or more times within a given season. This study received ethical approval from the Leeds Beckett University ethics committee, with informed consent obtained for participants over 16 years of age and parental consent for players under 16.

### 2.2 Procedures

The testing battery was composed of anthropometric measures, alongside gym and field‐based tests of physical qualities. Anthropometric measures included standing height, seated height, total body mass, and body composition. Gym‐based tests included the countermovement jump (CMJ) and isometric mid‐thigh pull (IMTP), whilst the field‐based tests included 40 m sprints, and a prone yo‐yo intermittent recovery test level 1 (prone Yo‐Yo IR1). Prior to the commencement of each testing session, players completed a warm‐up routine led by their club's strength and conditioning coach. These warm‐ups typically involved a series of dynamic stretches and bodyweight exercises such as squats and press ups prior to the gym‐based testing. Warm‐ups prior to the field‐based testing typically involved sub‐maximal running, dynamic stretching, and progressive sprint efforts building in distance and intensity.

#### 2.2.1 Anthropometry

Standing height was measured to the nearest 0.1 cm using a portable stadiometer (Seca 213, Hamburg, Germany). Body mass was measured to the nearest 0.1 kg and body fat percentage to the nearest 0.1%, both using a bioimpedance analyser (Tanita BF‐350, Tokyo, Japan). This device has shown an intra‐class correlation coefficient (ICC) of 0.93–0.98 when assessing body composition (Loenneke et al. 2013).

#### 2.2.2 Countermovement Jump

Participants completed two maximal effort CMJs on portable force plates (Passport Force Platform, PASCO Scientific, Roseville, CA; Lake et al. 2018). Jump height for each jump was recorded to the nearest 0.1 cm, with the peak value retained for analysis. Participants were instructed to drop to a self‐selected height before jumping as high as possible, without re‐bending their legs, and keeping their hands on their hips throughout (McMahon et al. 2022). Pasco Capstone software was used to estimate jump height based on flight time (Xu et al. 2023); this method has shown an ICC of 0.96–0.97 and a coefficient of variation (CV) of 2.93% (García‐López et al. 2013).

#### 2.2.3 Isometric Mid‐Thigh Pull

Participants completed two maximal effort IMTPs, measured in kilograms to the nearest 0.5 kg, with the peak value retained for analysis. The equipment and protocol used is outlined in Till et al. (2018), which has shown an ICC of 0.91 and CV of 6.0%. Peak force in newtons was estimated using a regression equation based on the value in kilograms (Till et al. 2018). Peak force relative to body mass was also calculated by dividing peak force in newtons by participants' total body mass in kilograms.

#### 2.2.4 Sprint Speed & Momentum

Participants performed two maximal effort 40 m sprints. Total time for each sprint and 10 m split times at intervals of 10, 20, 30, and 40 m, were recorded to the nearest 0.01 s, using Brower photoelectric timing gates (Brower Timing Systems, Draper, UT). Participants were required to start each sprint 0.5 m behind the start line in a two‐point stance, commencing at their own discretion (McCormack et al. 2021). Maximum mean velocity was estimated by dividing the 10 m split times by 10 m, with the peak value across both sprints retained for analysis. Ten‐metre mean sprint momentum was calculated by multiplying mean velocity over the first 10 m of each sprint by participants' body mass (McCormack et al. 2021), with the peak value from each sprint retained for analysis. The CV for 10, 20, 30, and 40 m split times have been reported as 2.5%, 2.2%, 2.2%, and 1.8%, respectively (Sawczuk et al. 2018).

#### 2.2.5 Prone Yo‐Yo Intermittent Recovery Test Level 1

The protocol for the prone Yo‐Yo IR1 is outlined in Dobbin et al. (2021). Participants' final level was determined when they failed their second shuttle or stopped running by choice, with the final successful level recorded as their score. The final level was converted to distance in metres by multiplying the number of successful levels by 40. The CV for the prone Yo‐Yo IR1 has been reported as 9.9% and the ICC as 0.98 (Dobbin et al. 2021).

### 2.3 Statistical Analysis

Latent growth curve models were used to assess differences in physical development between age groups (i.e., U15, U16, U17, U18; Curran et al. 2010). Models were created with the following physical qualities as the dependent variable; height, total body mass, lean body mass, IMTP peak force, relative IMTP peak force, CMJ height, 10 m sprint, 10 m sprint momentum, maximum velocity, and prone Yo‐Yo IR1 distance. Growth curves for each quality were created using the four testing time points; time point 1 (TP1) ‐ start of pre‐season, time point 2 (TP2) ‐ end of pre‐season, time point 3 (TP3)—mid‐season, and time point 4 (TP4) ‐ end of season.

Growth models were originally designed with ordinal loadings for each time point, meaning that the time periods between each time point are assumed to be equal (Geiser et al. 2013). The intercept loadings were fixed at 1, and the variance at each time point was unconstrained (Geiser et al. 2013). Age group was included as a categorical variable to assess the difference in growth curve trajectories. Missing data were estimated using the full information maximum likelihood method (Rosseel 2012). For the CMJ model dataset, TP4 was not included due to systematic missing data. For the maximum velocity model, the variance at each time point was constrained as the original model estimated negative variance at certain time points (Farooq 2022).

Slope coefficients for each age group represented the rate of development for each physical quality. These values indicate the estimated mean change between each time point for each age group (in the unit of measurement for that variable), with 95% confidence intervals (CIs) around the mean estimates. Intercepts and slope coefficients from each growth model were used to plot growth curves for each age group. Significant differences between age group slope coefficients were determined using *z* tests based on the differences in the mean estimates and associated standard errors (Paternoster et al. 1998). Between‐player variability was quantified as the mean difference between each player's slope coefficient and the mean slope coefficient (i.e., standard deviation) with 95% CIs. This was also converted to a CV by dividing the mean estimate by the standard deviation and multiplying by 100 (Weakley et al. 2023). Where CIs were reported for slope coefficients and between‐player variability, if the CIs included zero, there was deemed to be a lack of confidence in these estimates as the null hypothesis was not disproved (Sim and Reid 1999).

The chi squared statistic was used to assess model fit in relation to the covariance structure (Wu et al. 2009), whilst a robust estimate of root mean squared error of approximation (RMSEA) was used to assess model fit in relation to the marginal mean structure (Zhang and Savalei 2023). The thresholds for RMSEA interpretation were < 0.05 *good*, 0.05–0.08 *acceptable*, 0.08–0.10 *marginal*, > 0.1 *poor* (Kim et al. 2016). The Comparative Fit Index (CFI) was also used to assess model fit in relation to null and saturated models. It has previously been suggested that CFI values ≥ 0.95 indicate a good model fit; however, this should be considered in tandem with other model fit indices (Saskia van Laar 2021).

All statistical analyses were conducted in R Studio (V4.3.2, R Foundation for Statistical Computing, Vienna, Austria). Latent growth curve models were created using the *growth* function from the *lavaan* package (Rosseel 2012). This function also provided 95% confidence intervals for slope coefficients and model fit indices (RMSEA, Chi Squared, CFI).

## 3 Results

**FIGURE 1:**
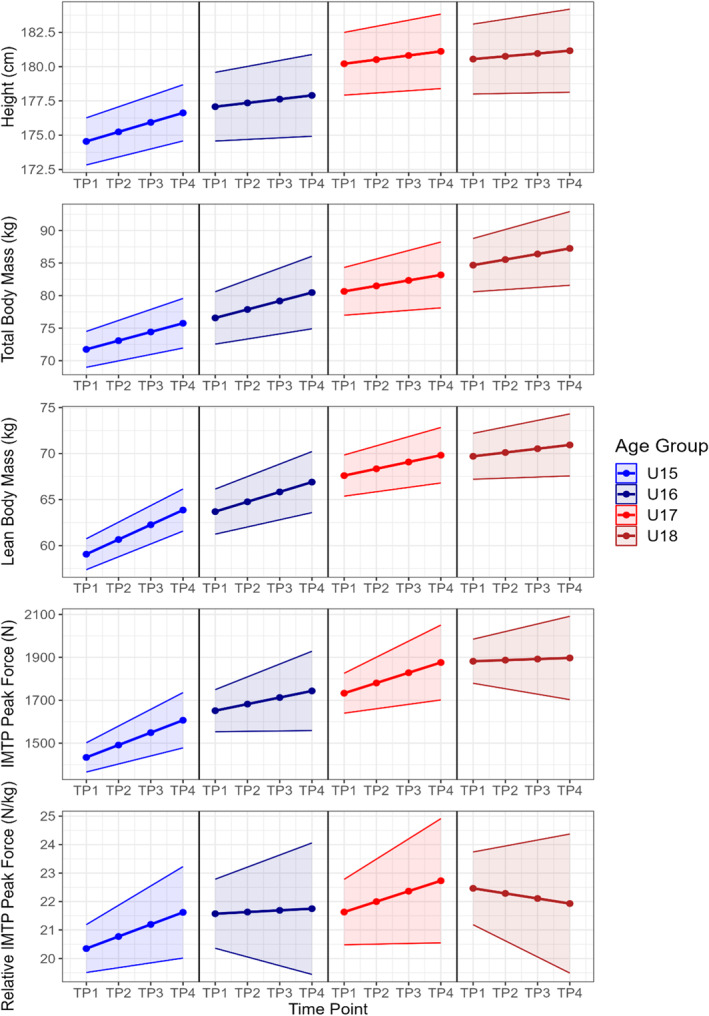
Estimated growth curves for each age group over the course of a season for height, total and lean body mass, IMTP peak force, and relative IMTP peak force. IMTP = isometric mid‐thigh pull, TP1 = time point 1, TP2 = time point 2, TP3 = time point 3, TP4 = time point 4.

**FIGURE 2:**
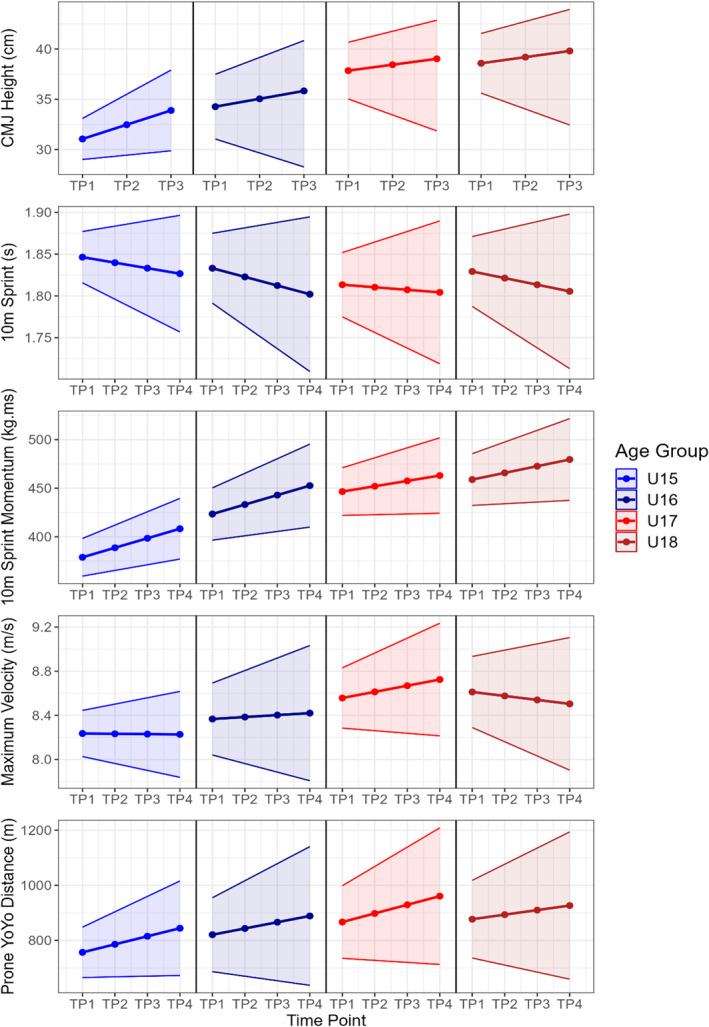
Estimated growth curves for each age group over the course of a season for CMJ height, 10 m sprint, 10 m sprint momentum, maximum velocity, and prone Yo‐Yo IR1 distance. CMJ = countermovement jump, TP1 = time point 1, TP2 = time point 2, TP3 = time point 3, TP4 = time point 4.

Figures 1 and 2 show the estimated growth curves for each physical quality across each age group, over the course of a season. The mean estimates at each time point from the latent growth curve models are presented with the shaded areas representing 95% CIs. Table 1 shows the rate of development for each physical quality across each age group, alongside the standard deviation in the rate of development for each quality due to between‐player variability. Model fit indices are available in Table S1.

**TABLE 1:** Rates of physical development between each time point for each physical quality by age group with 95% CIs and the coefficient of variation for between‐player variability in rates of development.

	U15 rate of development (95% CI)	U16 rate of development (95% CI)	U17 rate of development (95% CI)	U18 rate of development (95% CI)	Post‐hoc	Between‐player variability (95% CI)
Height (cm)	0.69 (0.58–0.81)	0.28 (0.12–0.43)	0.30 (0.16–0.45)	0.20 (0.05–0.32)	U15 > U16, U17, U18	0.26 (0.18–0.33)
Total body mass (kg)	1.34 (0.98–1.69)	1.30 (0.74–1.78)	0.84 (−0.12–0.80)	0.86 (−0.15–0.90)	U15 > U17	0.98 (0.78–1.14)
Lean body mass (kg)	1.60 (1.40–1.80)	1.07 (0.78–1.36)	0.74 (0.48–1.00)	0.41 (0.12–0.70)	U15 > U16, U17, U18	0.42 (0.20–0.55)
IMTP peak force (N)	57.7 (37.5–78.0)	30.7 (1.75–59.6)	47.7 (20.5–74.9)	5.08 (−25.4–35.6)	U15 > U18	27.8 (−28.7–48.7)
Relative IMTP peak force (N/kg)	0.42 (0.17–0.68)	0.06 (−0.31–0.42)	0.37 (0.02–0.71)	−0.18 (−0.57–0.21)	U15 > U18	0.28 (−0.41–0.56)
CMJ height (cm)	1.42 (0.43–2.41)	0.78 (−0.75–2.31)	0.59 (−0.75–1.93)	0.61 (−0.78–2.00)		1.36 (−1.74–2.60)
10 m sprint (s)	−0.007 (−0.020–0.006)	−0.011 (−0.028–0.006)	−0.003 (−0.019–0.012)	−0.008 (−0.025–0.009)		0.017 (−0.002–0.024)
10 m sprint momentum (kg.ms)	9.80 (5.84–13.8)	9.75 (4.48–15.01)	5.50 (0.73–10.28)	6.89 (1.74–12.03)		5.93 (2.41–8.03)
Maximum velocity (m/s)	−0.003 (−0.063–0.057)	0.017 (−0.078–0.113)	0.055 (−0.024–0.134)	−0.036 (−0.129–0.057)		0.03 (−0.10–0.32)
PYIR1 distance (m)	29.17 (2.46–55.88)	22.69 (−16.49–61.87)	31.31 (−7.41–70.02)	16.53 (−25.58–58.64)		39.0 (−23.8–60.1)

Abbreviations: CI = confidence interval, CMJ = countermovement jump, IMTP = isometric mid‐thigh pull, PYIR1 = prone Yo‐Yo intermittent recovery test level‐1.

### 3.1 Anthropometrics

Height was estimated to increase in all age groups, with progressively higher values seen in older age groups (Figure 1). The rate of development in height varied by age group, with U15s increasing height significantly faster than all other age groups (*p* < 0.001; Table 1). Total and lean body mass were also estimated to increase in all age groups, with progressively greater total and lean body mass values estimated for older age groups (Figure 1). The U15s developed total body mass at the fastest rate, which was significantly faster than the U17s (*p* = 0.045). The difference between the U15s and U18s also approached significance (*p* = 0.066; Table 1). Lean body mass also developed at a significantly faster rate in the U15s than all other age groups (*p* < 0.001; Table 1). Large between‐player variability was evident for height, total, and lean body mass. The between‐player CV for height was 93% in U16s, 87% for U17s, and 130% for U18s. For total body mass the between‐player CV was 117% for U17s and 114% for U18s, whilst for lean body mass it was 102% in the U18s.

### 3.2 Isometric Mid‐Thigh Pull

IMTP peak force was estimated to increase for all age groups, with progressively higher values estimated in older age groups (Figure 1). The U15s developed IMTP peak force significantly faster than U18s (*p* = 0.002), whilst the difference between U15s and U16s approached significance (*p* = 0.07). Relative IMTP peak force was estimated to increase in all age groups except the U18s (Figure 1). The U15s developed relative IMTP peak force significantly faster than U18s (*p* = 0.006), whilst the difference between the U15s and U16s approached significance (*p* = 0.054).

### 3.3 Speed, Power, Momentum, and Cardiovascular Fitness

Sprint momentum was estimated to increase within each age group, with between‐player CVs of 61%–107% dependent on age group. Confidence intervals for CMJ height, 10 m sprint, maximum velocity, and prone Yo‐Yo IR1 distance overlapped zero for rates of development in several age groups, suggesting a lack of confidence in any age group‐related trends in the development of these qualities. No significant differences were identified between age groups for rate of development in CMJ height, 10 m sprint, 10 m sprint momentum, maximum velocity, or prone Yo‐Yo IR1 distance.

## 4 Discussion

This study aimed to compare rates of physical development between age groups in youth rugby league players from multiple clubs within the professional rugby league TIDS. Between‐player differences in rates of physical development were also analysed. Findings showed that each age group increased body size, strength, and sprint momentum, with younger age groups developing body size and strength at a faster rate. However, substantial between‐player variation was evident in the development of body size and sprint momentum. Age group‐related differences in the development of CMJ height, sprint speed, and cardiovascular fitness were not apparent in this study. These findings suggest that early talent identification decisions in the rugby league TIDS may be ineffective due to the ongoing, individually variable development of body size, strength, and sprint momentum.

This study highlighted the U15 age group as a key phase for the development of strength. Findings showed that U15s developed strength at a significantly faster rate than all other age groups. This supports existing literature using single‐club samples, which showed greater seasonal improvements in upper and lower body strength in U15s and U16s compared to older age groups (Till et al. 2015, 2014). Age‐group related differences in strength development may stem from the U15 age group being many players' first exposure to structured strength and conditioning training (Coutts et al. 2004), as it is the start point of the rugby league TIDS. Indeed, U15 players complete up to 151 min per week of gym‐based training in the TIDS (McCormack et al. 2020), likely a substantial increase on their previous exposure to this type of training. In support of this, movement competency was found to be significantly higher in older compared with younger players and correlated with strength qualities in youth players across age groups (Ireton et al. 2019). This suggests that faster rates of strength development in U15s in this study may partially be explained by increases in movement competency and resistance training technique resulting from structured strength and conditioning training (Coutts et al. 2004). Alternative factors should also be considered when attempting to understand differences in rates of strength development, given that research regarding the relationship between training age and physical development in youth rugby league players is equivocal (Booth et al. 2020; Till, Darrall‐Jones, et al. 2017). Ultimately, findings from this study suggest that early talent identification decisions made upon entry into the TIDS may be confounded by the increases in strength seen in U15 players. This is particularly relevant given the perceived importance of strength to youth players' performance (McCormack et al. 2020) and career attainment (Till et al. 2016).

Findings from this study also showed that U15s developed total and lean body mass at a significantly faster rate than older age groups. This may also result from potential increases in U15 players' resistance training load. However, between‐player CVs exceeding 70% were seen for total and lean body mass, suggesting that individual player characteristics such as chronological or relative age, and biological maturation may be creating variability in rates of development for body mass (Owen et al. 2022; Till et al. 2013). Whilst this study did not directly assess biological maturation, height was also found to develop at a significantly faster rate in U15s compared with older age groups. Given that height typically develops in‐line with biological maturation, this suggests that the effects of growth and maturation are more pronounced in the U15 age group. Seasonal increases in body mass have been evidenced previously across age groups in youth rugby league players (Till et al. 2015), whilst future professionals have been shown to increase body mass more quickly than their peers (Till et al. 2016). As such, monitoring body mass is recommended as part of the talent identification and development processes (Till et al. 2015, 2014; Till et al. 2016). However, practitioners should consider individual player characteristics when monitoring body mass to understand individual and age‐group differences in rates of body mass development.

Youth rugby league players are typically earlier maturing than the general population. Previous evidence shows that European males exhibit an age of peak height velocity of 14.2 years, compared with 13.6 years in youth rugby league players (Till et al. 2017). This notion is reinforced by comparisons to the World Health Organisation growth charts (Moy and Wright 2014), which show that participants in this study developed body mass at a slower rate in younger age groups (i.e., U15s and U16s), but a faster rate in older age groups (i.e., U17s and U18s), than weight‐matched members of the general population. When comparing to other sports, U15 players in this study were estimated to increase body mass by 5.6% over a season, which is lower than age‐matched soccer players (7.4%; Morris et al. 2018), basketball players (13%; te Wierike et al. 2014), and hockey players (10%; Elferink‐Gemser et al. 2007). Furthermore, U15 players in this study increased strength by 12.1% over a season, which was less than the 16.6% seasonal increase seen in U15 soccer players (Morris et al. 2018). These differences between sports may be explained by rugby league players maturing earlier, as indicated by smaller increases in height compared with other sports (Elferink‐Gemser et al. 2007; te Wierike et al. 2014; Morris et al. 2018), although this does not represent a direct measurement of biological maturation. Nonetheless, growth and maturation causes increases in anabolic growth hormones (Jansson et al. 2024), which could explain the faster rates of body mass and strength development seen in the U15s (Almeida‐Neto et al. 2020). This body of evidence suggests that early talent identification decisions may be biased by maturation status because of its relationship with strength and size. This advocates the use of methods such as maturity offset equations to inform early talent identification and selection decisions in youth rugby league players (Till and Jones 2015).

Limited resources within TIDS often necessitate potentially ineffective early talent identification and (de)selection decisions (Bailey and Collins 2013). These decisions are hindered by the unpredictable and individually variable nature of talent development in youth athletes (Baker et al. 2018; Till and Baker 2020; Till et al. 2013). The between‐player variability in the development of body size and sprint momentum evident in this study suggest that predicting physical development is likely to be challenging, which can limit the effectiveness of any talent identification and (de)selection decisions made in earlier age groups. Furthermore, the links between maturation, body size, and strength qualities suggests that early talent identification and selection decisions may be biased towards earlier maturing players in younger age groups even though their physical advantages may be transient (Till et al. 2013). Increased strength and size are also associated with enhanced tackling and ball‐carrying ability in youth players (Gabbett et al. 2010; Waldron et al. 2014), indicating that enhanced physical qualities, as a result of earlier maturation, may facilitate on‐field performance and thus further influence talent identification and selection decisions. As such, delaying talent identification decisions by altering the start point of the TIDS can encourage the inclusion and retention of more talented players in the TIDS. Delaying the start point of the TIDS would also allow players to delay sport specialisation and potentially sample a wider range of sports and experience more varied training. This could reduce injury risk and facilitate players' long‐term development in the sport (McLellan et al. 2022). Certainly, senior professional players have exhibited varied trajectories as youth players in terms of the point at which they began to specialise in the sport (Cupples et al. 2018), indicating that later specialisation would not necessarily inhibit players' career attainment.

The rate of development for other physical qualities in this study did not differ between age groups; CMJ height, 10 m sprint, maximum velocity, 10 m sprint momentum, and prone Yo‐Yo IR1 distance showed no significant differences between age groups. This may be a result of the consistent increases in total body mass seen in each age group, which has been suggested to reduce the development of locomotor activities in youth players (Till et al. 2014). This is reflected in the consistent increases in 10 m sprint momentum, but not 10 m sprint times, evident in each age group. As such, the development of locomotor qualities such as sprint speed and prone Yo‐Yo performance may not become evident until later age groups where the rate of body mass development is reduced. This notion is supported by evidence showing that older players (U18s and U20s) have also previously shown greater improvements in these qualities than those in younger age groups (Till et al. 2014). These findings are in contrast to evidence in youth soccer showing that players exhibit a consistent increases from 11 to 17 years of age in CMJ and 10 m sprint performance, alongside linear increases in body mass (Dugdale et al. 2024). These contrasting findings may be explained by the substantially lower body mass exhibited by the youth soccer players, whereby they require less well‐developed lower body speed and power qualities to overcome their body mass when sprinting (Samozino et al. 2016). Nonetheless, the lack of age‐group differences in the development of these qualities suggests that early talent identification and selection decisions based around these qualities may be more valid as higher performing players are likely to retain their advantages in older age groups. Previous work has shown sprint speed to correlate with number of tries scored in senior players (Gabbett et al. 2011a), whilst enhanced lower body power and sprint acceleration appear related to superior tackling proficiency in youth players (Gabbett et al. 2010), supporting the notion that basing talent identification and selection decisions around speed and power qualities may be more effective in earlier age groups than body size and strength qualities.

This study provides insights into age group‐based differences in physical development which can inform the structure of the rugby league TIDS. This study is also the first, to the authors' knowledge, to analyse differences in physical development between age groups using a multi‐club sample, thus increasing the generalisability of the results to the wider TIDS population. This study also builds on previous work by assessing players at multiple time points within each season to account for potential variation in rates of change between time points (Carron et al. 2024; Till et al. 2015, 2014). However, this study was not without its limitations. A primary issue was the model fit in relation to the marginal mean structure, whereby RMSEA values for most models were marginal to poor (Table S1). This suggests that marginal mean values at each time point may not be representative of actual data (Wu et al. 2009). Despite this, significant Chi‐squared values were seen for several models, alongside CFI values ≥ 0.95, suggesting the overall fit of the models to the data was good (Table S1) (Saskia van Laar 2021; Wu et al. 2009). Furthermore, longitudinal data collection in this study was limited to one season per player. Future studies should aim to collect repeated measures data over multiple seasons to provide a true representation of the longitudinal development of youth rugby league players.

## 5 Conclusions

This study highlighted the U15 age group as a key phase for the development of body size and strength qualities in youth rugby league players. These qualities increased within each age group; however, substantial between‐player variation was evident. Natural growth and maturation may play a role in the development of body size and strength qualities, indicating that delaying talent identification and selection decisions reduces the risk of bias towards earlier maturing players. However, CMJ height, sprint speed, and performance on the prone Yo‐Yo IR1 showed no differences in their development based on age. As such, utilising these measures during the talent identification and selection process may be more effective in earlier age groups. The national governing body should consider how best to design and structure the TIDS to facilitate effective talent identification, selection, and development. Results suggest that delaying the start point could enhance the effectiveness of talent identification and selection decisions which may increase the retention of talent within the TIDS.

## Funding

This study was supported by Rugby Football League.

## Conflicts of Interest

The authors declare no conflicts of interest.

## Supporting information


**Table S1**: Model fit statistics. RMSEA = root mean squared error of approximation, *p < 0.05, **p < 0.01, ***p < 0.001.
