# Supplementary material for: Can the Physical Development Trajectories of Rugby League Players at Different Age Groups Inform the Talent Pathway? A Multi‐Club Study of 261 Players
Source: Eur J Sport Sci. 2025 Dec 8;26(1):e70100. doi: 10.1002/ejsc.70100 (PMC12685470; doi:10.1002/ejsc.70100)
Supplement: Supplementary file 1 — Table S1: Model fit statistics. RMSEA = root mean squared error of approximation, *p < 0.05, **p < 0.01, ***p < 0.001. [file EJSC-26-e70100-s001.docx]

# **Table S1**

Supplemental Table 1 - Model fit statistics. RMSEA=root mean squared error of approximation, *p<0.05, **p<0.01, ***p<0.001.

|  | RMSEA | Chi Squared | CFI |
| --- | --- | --- | --- |
| Height | 0.12 *poor* | 30.56^**^ | 0.99 |
| Total body mass | 0.08 *acceptable* | 19.29 | 1.00 |
| Lean body mass | 0.10 *marginal* | 27.76^**^ | 0.99 |
| IMTP peak force | 0.15 *poor* | 46.19^***^ | 0.95 |
| Relative IMTP peak force | 0.16 *poor* | 52.56^***^ | 0.91 |
| CMJ height | 0.11 *poor* | 8.84 | 0.98 |
| 10 m sprint | 0.11 *poor* | 30.77^**^ | 0.91 |
| 10 m sprint momentum | 0.10 *marginal* | 19.42 | 0.99 |
| Maximum velocity | 0.23 *poor* | 53.61^***^ | 0.83 |
| PYIR1 distance | 0.19 *poor* | 44.59^***^ | 0.91 |

RMSEA=root mean square error of approximation, CFI=comparative fit index, IMTP=isometric mid-thigh pull, CMJ=countermovement jump, PYIR1=prone Yo-Yo intermittent recovery test level-1.
